# Supplementary material for: Proteomics biomarker discovery for individualized prevention of familial pancreatic cancer using statistical learning
Source: PLoS One. 2023 Jan 26;18(1):e0280399. doi: 10.1371/journal.pone.0280399 (PMC9879447; doi:10.1371/journal.pone.0280399)
Supplement: S1 Fig — Averaged ROC curves of the prediction performance adaptive lasso (yellow), glmboost (blue), and gamboost (orange) are estimated based on 40 subsamples generated by the repeated stratified 4-fold cross-validation in the three scenarios of the FaPaCa study. The shaded areas represent the one standard deviation intervals. At the right bottom corner, the averaged AUCs and their standard deviations are shown. (DOCX) [file pone.0280399.s001.docx]

| 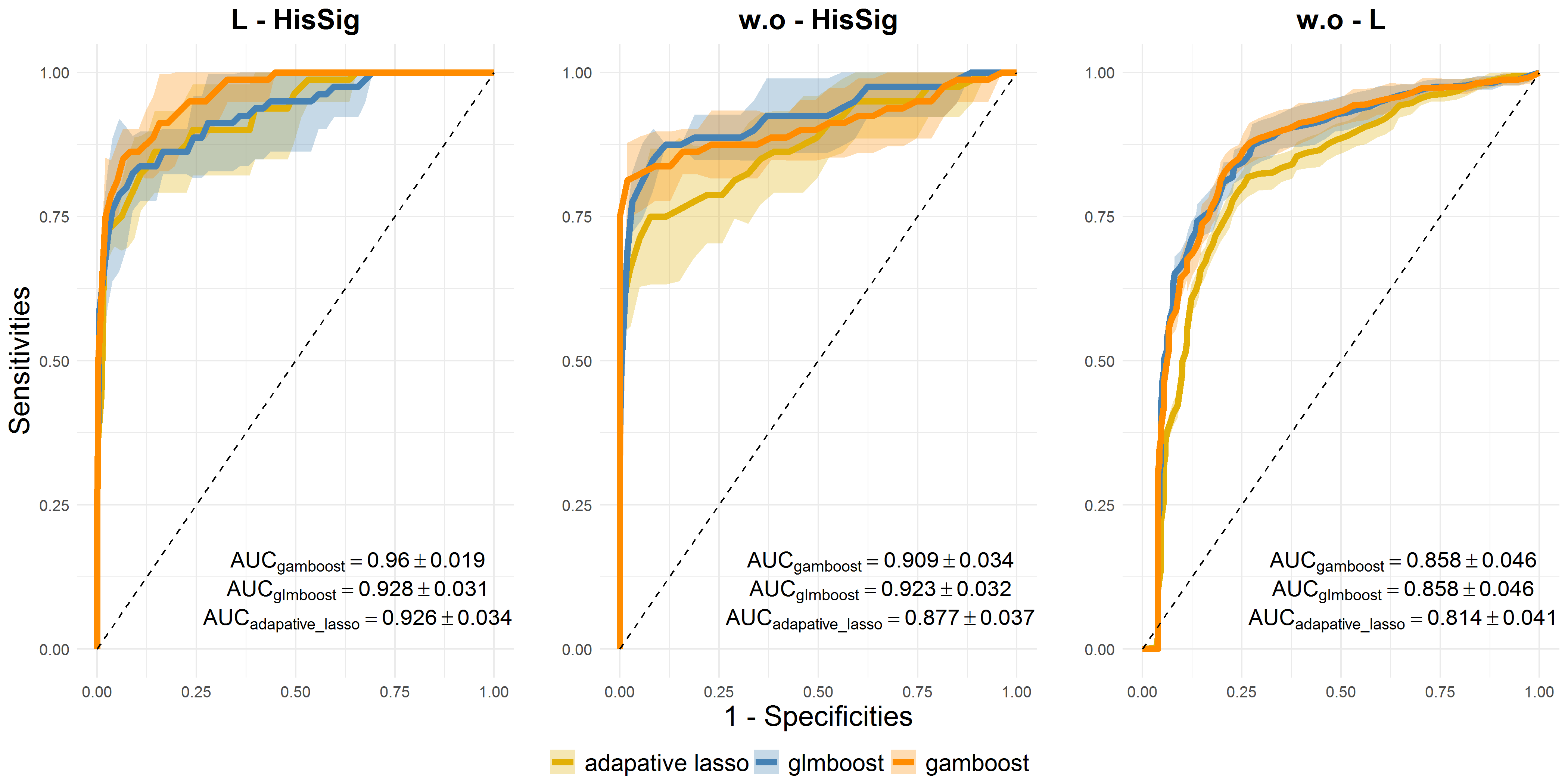 |
| --- |
| **S1 Fig. Averaged ROC curves of the prediction performance adaptive lasso, glmboost, and gamboost.** Averaged ROC curves of the prediction performance adaptive lasso (yellow), glmboost (blue), and gamboost (orange) are estimated based on 40 subsamples generated by the repeated stratified 4-fold cross-validation in the three scenarios of the FaPaCa study. The shaded areas represent the one standard deviation intervals. At the right bottom corner, the averaged AUCs and their standard deviations are shown. |
