# Supplementary material for: Proteomics biomarker discovery for individualized prevention of familial pancreatic cancer using statistical learning
Source: PLoS One. 2023 Jan 26;18(1):e0280399. doi: 10.1371/journal.pone.0280399 (PMC9879447; doi:10.1371/journal.pone.0280399)
Supplement: S2 Fig — Each boxplot summarizes the results of 40 subsamples generated by repeated stratified 4-fold cross-validation in each fold in the respective scenario L-HisSig (red), w.o-HisSig (green) and w.o-L (yellow). (DOCX) [file pone.0280399.s002.docx]

| 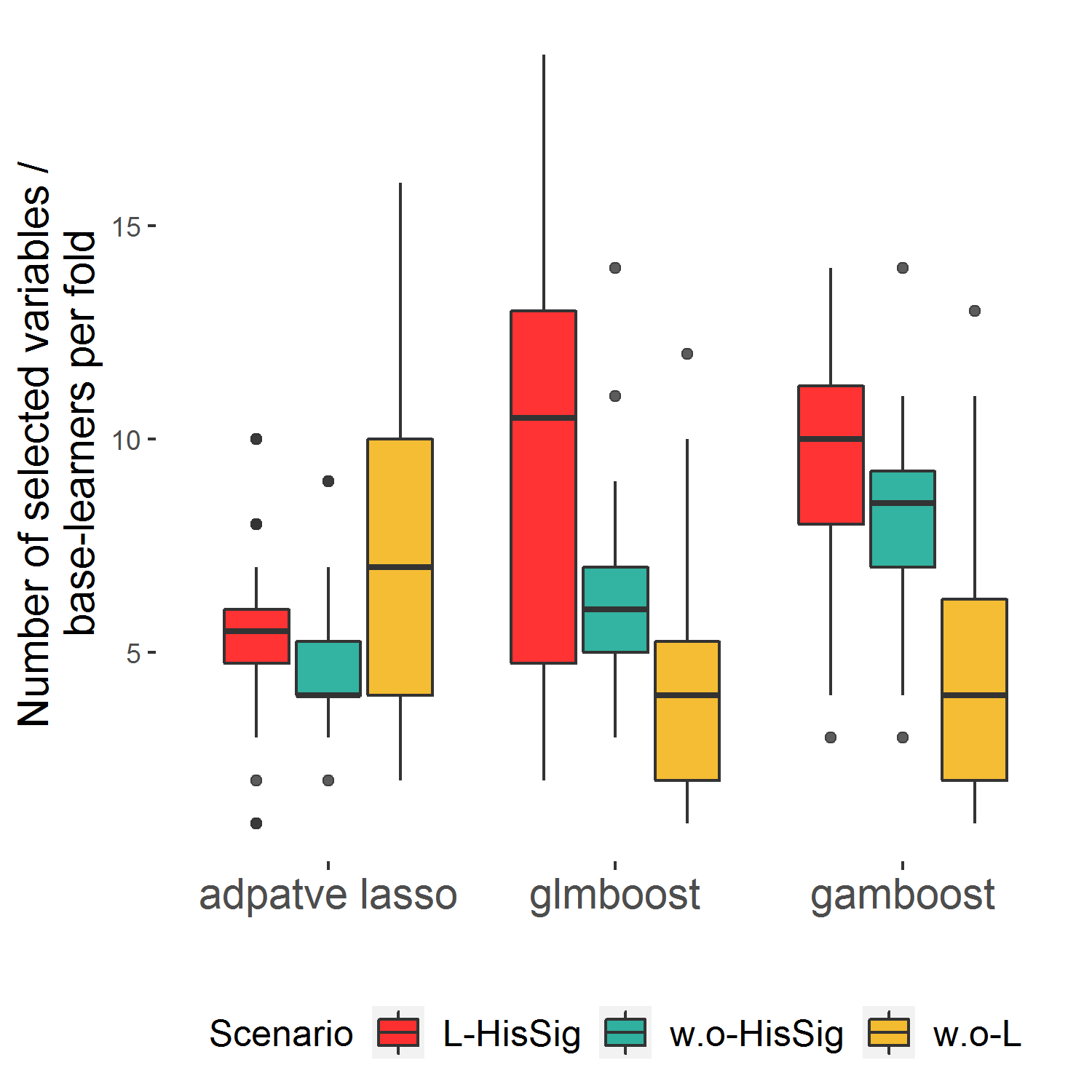 |
| --- |
| **S2 Fig. Number of selected variables/base-learners by adaptive lasso, glmboost, and gamboost.** Each boxplot summarizes the results of 40 subsamples generated by repeated stratified 4-fold cross-validation in each fold in the respective scenario L-HisSig (red), w.o-HisSig (green) and w.o-L (yellow). |
