# Supplementary material for: Proteomics biomarker discovery for individualized prevention of familial pancreatic cancer using statistical learning
Source: PLoS One. 2023 Jan 26;18(1):e0280399. doi: 10.1371/journal.pone.0280399 (PMC9879447; doi:10.1371/journal.pone.0280399)
Supplement: S4 Fig — The stability selection results using adaptive lasso (bottom) and glmboost (top) among the scenarios L-HisSig (red), w.o-HisSig (green), and w.o-L (yellow). The grey line represents the corresponding cut-off level under the assumption of a unimodal distribution. The assays with selection probability higher than (black font) or slightly below the cut-off (red font) are annotated. (DOCX) [file pone.0280399.s004.docx]

| 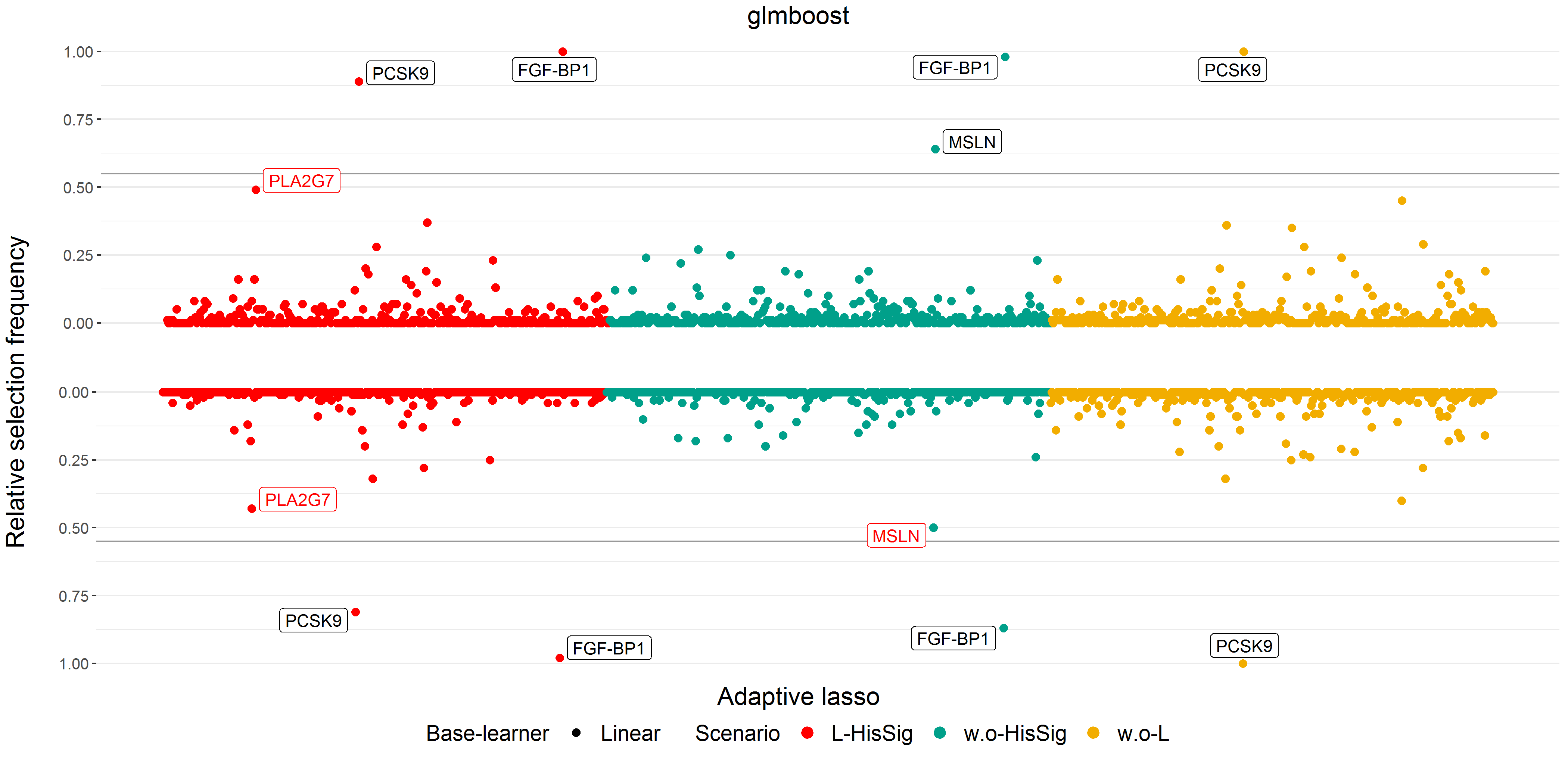 |
| --- |
| **S4 Fig. Summary of the stability selection results using adaptive lasso and glmboost.** The stability selection results using adaptive lasso (bottom) and glmboost (top) among the scenarios L-HisSig (red), w.o-HisSig (green), and w.o-L (yellow). The grey line represents the corresponding cut-off level under the assumption of a unimodal distribution. The assays with selection probability higher than (black font) or slightly below the cut-off (red font) are annotated. |
