# Supplementary material for: Proteomics biomarker discovery for individualized prevention of familial pancreatic cancer using statistical learning
Source: PLoS One. 2023 Jan 26;18(1):e0280399. doi: 10.1371/journal.pone.0280399 (PMC9879447; doi:10.1371/journal.pone.0280399)
Supplement: S6 Fig — (A) depicts the prediction results of the fitted gamboost model. (B), (C), and (D) represent the mean, lower quantile (2.5%), and upper quantile (97.5%) of the prediction results estimated via bootstrapping. (DOCX) [file pone.0280399.s006.docx]

| 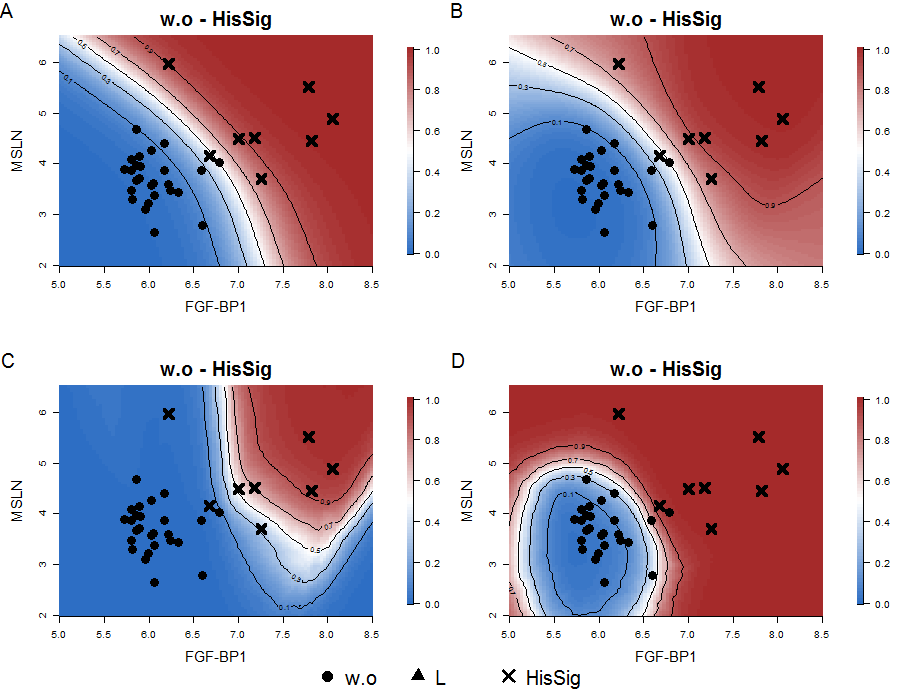 |
| --- |
| **S6 Fig. The estimated classification results of gamboost in scenario w.o-HisSig.** (A) depicts the prediction results of the fitted gamboost model. (B), (C), and (D) represent the mean, lower quantile (2.5%), and upper quantile (97.5%) of the prediction results estimated via bootstrapping. |
