# Supplementary material for: Proteomics biomarker discovery for individualized prevention of familial pancreatic cancer using statistical learning
Source: PLoS One. 2023 Jan 26;18(1):e0280399. doi: 10.1371/journal.pone.0280399 (PMC9879447; doi:10.1371/journal.pone.0280399)
Supplement: S3 Table — (DOCX) [file pone.0280399.s009.docx]

**S3 Table. Results of over-representation analysis using Reactome pathway database**

| Pathway ID | Description | Gene Ratio | Background Ratio | p-value | Adjusted p-value | q-value | Involved proteins/genes | References |
| --- | --- | --- | --- | --- | --- | --- | --- | --- |
| R-HSA-163125 | Post-translational modification: synthesis of GPI-anchored proteins | 2/5 | 6/286 | 0.00358 | 0.03301 | 0.00965 | MSLN/LYPD3 | - |
| R-HSA-392499 | Metabolism of proteins | 4/5 | 50/286 | 0.00367 | 0.03301 | 0.00965 | PCSK9/MSLN/PLA2G7/LYPD3 | - |
| R-HSA-597592 | Post-translational protein modification | 3/5 | 35/286 | 0.01421 | 0.03497 | 0.01022 | PCSK9/MSLN/LYPD3 | - |
| R-HSA-190236 | Signaling by FGFR | 1/5 | 1/286 | 0.01748 | 0.03497 | 0.01022 | FGFBP1 | Dailey et al. 2005, Beenken et al. 2009 |
| R-HSA-190241 | FGFR2 ligand binding and activation | 1/5 | 1/286 | 0.01748 | 0.03497 | 0.01022 | FGFBP1 | Zhang et al. 2006, Moffa et al. 2007 |
| R-HSA-190377 | FGFR2b ligand binding and activation | 1/5 | 1/286 | 0.01748 | 0.03497 | 0.01022 | FGFBP1 | Zhang et al. 2006, Moffa et al. 2007 |
| R-HSA-422085 | Synthesis, secretion, and deacylation of Ghrelin | 1/5 | 1/286 | 0.01748 | 0.03497 | 0.01022 | PLA2G7 | Soares et al. 2008, Yin et al. 2009 |
| R-HSA-5654738 | Signaling by FGFR2 | 1/5 | 1/286 | 0.01748 | 0.03497 | 0.01022 | FGFBP1 | Dailey et al. 2005, Beenken et al. 2009 |
| R-HSA-8866427 | VLDLR internalisation and degradation | 1/5 | 1/286 | 0.01748 | 0.03497 | 0.01022 | PCSK9 | Poirier et al. 2008 |
