# Supplementary material for: Proteomics biomarker discovery for individualized prevention of familial pancreatic cancer using statistical learning
Source: PLoS One. 2023 Jan 26;18(1):e0280399. doi: 10.1371/journal.pone.0280399 (PMC9879447; doi:10.1371/journal.pone.0280399)
Supplement: S1 Appendix — (DOCX) [file pone.0280399.s010.docx]

## S1 Appendix - Supplementary methods

## Ridge regression

In the context of high dimensional data, i.e., p > n, no unique solution is found for ordinary linear regression due to the underdetermined linear system. The ridge regression model is a standard statistical remedy by introducing an L2-penalty term into the regression model. The penalty term induces a shrinkage effect to the estimated regression coefficients, controlled by a tuneable non-zero shrinkage hyperparameter $\lambda$. Cross-validation is the most common method for searching the optimal value of $\lambda$. With the help of the penalty term, the ridge regression model enables the inclusion of all the features in the statistical learning process.

|  | $\hat{\beta}(ridge)=\underset{\beta}{\text{argmin}}\left\Vert y-\sum_{j=1}^{p} \mathbf{x}_{j}\beta_{j} \right\Vert^{2}+\lambda\sum_{j=1}^{p} \parallel\beta_{j}\parallel_{2}$ | (S1-1) |
| --- | --- | --- |

## Adaptive lasso

Lasso regression is one of the most popular variable selection models owing to its simultaneous model fitting and variable selection nature. Lasso estimates are defined as

|  | $\hat{\beta}(lasso)=\underset{\beta}{\text{argmin}}\left\Vert y-\sum_{j=1}^{p} \mathbf{x}_{j}\beta_{j} \right\Vert^{2}+\lambda\sum_{j=1}^{p} \parallel\beta_{j}\parallel_{1}$ | (S1-2) |
| --- | --- | --- |

The L1-penalty term shrinks the estimated coefficients towards zero. When lambda is sufficiently large, variable selection is implemented automatically by further shrinking the coefficients to precisely zero. The resulting non-zero variables are, in turn, regarded as the active variable subset. This compelling variable selection property led to high recognition of the classical lasso regression in statistical learning. Nevertheless, one of the limitations of the classical lasso is its inconsistency in variable selection, especially when $p>n$. Zou 2006 [1] proposed the adaptive lasso, an improved version of classical lasso, aiming at a consistent variable selection.

Instead of a uniform penalization across the coefficients, a variable-specific weight vector is introduced to the L1-penalty term. The adaptive weights support the oracle properties of the adaptive lasso that the variables with lighter weights are more likely selected into the final model. The variable-specific weights stabilize the variable selection procedure of lasso and enhance the consistency of selected variables.

|  | $\hat{\beta}(adaptive lasso)=\underset{\beta}{\text{argmin}}\left\Vert y-\sum_{j=1}^{p} \mathbf{x}_{j}\beta_{j} \right\Vert^{2}+\lambda_{n}\sum_{j=1}^{p} \hat{w_{j}}\parallel\beta_{j}\parallel_{1}$ | (S1-3) |
| --- | --- | --- |

In the case of high dimensional data or potential collinearity, Zou 2006 suggested using the estimated coefficients $\hat{\beta}(ridge)$ from the best ridge regression fit as the weight vector $\hat{w_{j}}$.

|  | $\begin{matrix} \hat{w_{j}}=\left( \frac{1}{\left\vert\hat{\beta}\left( ridge \right) \right\vert} \right)^{\gamma} & where \gamma>0 \end{matrix}$ | (S1-4) |
| --- | --- | --- |

## Model-based gradient boosting

Along with regularised regression models, ensemble learning is also one of the popular approaches in ’omics studies. The boosting algorithm is one of the ensemble methods in machine learning, iteratively combining some weak base learners into a strong learner [2]. Statistical boosting combines the boosting algorithm with the classical generalized additive model (GAM) [3]–[5]. The statistical structure of the GAM makes the boosted model more interpretable than the tree-based boosting models [4]. Several biomedical studies have successfully applied statistical boosting to identify informative biomarkers [6], [7]. In this study, we focus on applying the gradient boosting approach, also named model-based gradient boosting (mboost), to a binary classification problem.

Mboost uses the GAM as the fundamental modelling structure. In our study, the expected binary outcome $Status$ given the observed value of predictors $X$, such as sex, age, and protein biomarkers, is modelled using the logit link function $g$:

| $g\left( \mathbb{E}\left( Status \vert X \right) \right)=\beta_{0}+f_{1}\left( Sex \right)+f_{2}\left( Age \right)+$  $f_{3}(Biomarker_{1})+\ldots+f_{p+2}(Biomarker_{p})$ | (S1-5) |
| --- | --- |

The function $f_{i}$ is called the base-learner of the corresponding predictor. The flexible additive structure of GAM allows us to include a wide range of predictor effects into the model, such as linear effects, smoothing non-linear effects using regression splines, or random effects [8], [9]. In this study, we are interested in the linear and potential non-linear effects of the biomarkers. Hence, we followed the modelling technique suggested by Hofner et al. 2014 and introduced both the linear base-learner and the centered smoothing p-spline base-learner for each biomarker [10]. These two base-learners can capture the linear trend effects and non-linear effects of the biomarkers independently.

In the gradient boosting approach, a loss function $\rho(y,f(x))$ must be specified before the model fitting [2], [4]. In the GAM model class, the loss function is typically the negative log-likelihood of the corresponding distribution of the conditionally expected outcome. For example, the negative binomial log-likelihood is the loss function in the binary classification problem:

|  | $\rho(y,f(x))=-\frac{1}{N}\sum_{i=1}^{N} {[y}_{i}\cdot log(g^{-1}(f(x)))-(1-y_{i})\cdot log(1-g^{-1}(f(x)))]$ | (S1-6) |
| --- | --- | --- |

where $g^{-1}$ is the inverse logit function. In each boosting iteration, all base-learners are individually fitted to the negative gradient of the loss function, evaluated in the previous iteration. Then, only the best-fitted base-learner $f_{i}$ is selected according to the residual sum of squares of the model, and the fitted model is updated by $f_{i}\cdot\nu$, where $\nu$ is the learning rate. The iterative fitting process will stop after a specified number of iterations $m_{stop}$. The algorithm of mboost has been described in detail by Mayr et al. 2017 [3].

Apart from the architecture of the base-learners, $m_{stop}$ and $\nu$ are the only hyperparameters in mboost. The choice of $m_{stop}$ crucially affects the performance of mboost, especially with regard to the danger of overfitting. We followed the recommendations of Mayr et al. 2012 and took the AIC-estimated $m_{stop}$ as a reference for the estimation of the optimal $m_{stop}$ via grid search to reduce the computational cost [11]. For the learning rate $\nu$, Schmid and Hothorn 2008 recommended keeping $\nu$ at a low value, e.g., $\nu=0.1$ [8].

## Stability selection

Stability selection, proposed by Meinshausen and Bühlmann [12], is an effective approach designed to enhance the performance of the variable selection process by controlling the number of falsely selected variables. Furthermore, the flexible algorithmic framework of stability selection works seamlessly with all variable selection methods.

The general idea of stability selection is to randomly divide the dataset into two equal halves and then perform variable selection with a predetermined algorithm on one half. The model fitting process is fine-tuned and then stopped when a subset of variables $\hat{S}^{\left\lfloor n/2 \right\rfloor}$ (or base-learners in context of mboost) of a pre-specified size $q$ has been selected. By repeating this procedure $B$ times, e.g., $B=100$, the results can be summarized with the cumulated selection frequency of each variable $\hat{\pi}_{j}$.

|  | $\hat{\pi}_{j}=\sum_{b=1}^{B} \mathbb{I}_{\{j\in\hat{S}_{b}^{\lfloor n/2\rfloor}\}}$ | (S1-7) |
| --- | --- | --- |

The so-called subset of stable variables is defined as the variables with a selection frequency above a prespecified threshold $\pi_{thr}$. The stability selection can control the per-family error rate ($PFER$) by providing the upper bound of the expected number of false-positive selections ($V$) :

|  | $E(V)\leq\frac{q^{2}}{(2\pi_{thr}-1)\cdot p}$ | (S1-8) |
| --- | --- | --- |

where $p$ is the total number of variables. The threshold $\pi_{thr}$ can be defined a priori or calculated by fixing $q$ via equation S1-8. This approach is typically helpful for researchers to monitor false discoveries when analyzing high dimensional data.

Later, Shah and Samworth proposed a modified version, complementary pairs stability selection ($CPSS$), to ease the required strong exchangeability assumption of irrelevant variables [13]. Instead of only using one random half of the data in each iteration, both of the divided subsamples, i.e. both “complementary pairs”, are included in the stability selection procedure. The authours further derived three error bounds without the need for an exchangeability assumption. In this study, we implemented CPSS on top of adaptive lasso and mboost.

References:

[1] H. Zou, ‘The Adaptive Lasso and Its Oracle Properties’, *J. Am. Stat. Assoc.*, vol. 101, no. 476, pp. 1418–1429, Dec. 2006, doi: 10.1198/016214506000000735.

[2] P. Bühlmann and T. Hothorn, ‘Boosting Algorithms: Regularization, Prediction and Model Fitting’, *Stat. Sci.*, vol. 22, no. 4, Nov. 2007, doi: 10.1214/07-STS242.

[3] A. Mayr, B. Hofner, E. Waldmann, T. Hepp, S. Meyer, and O. Gefeller, ‘An Update on Statistical Boosting in Biomedicine’, *Comput. Math. Methods Med.*, vol. 2017, p. 6083072, 2017, doi: 10.1155/2017/6083072.

[4] A. Mayr, H. Binder, O. Gefeller, and M. Schmid, ‘The evolution of boosting algorithms. From machine learning to statistical modelling’, *Methods Inf. Med.*, vol. 53, no. 6, pp. 419–427, 2014, doi: 10.3414/ME13-01-0122.

[5] T. Hastie and R. Tibshirani, ‘Generalized Additive Models’, *Stat. Sci.*, vol. 1, no. 3, Aug. 1986, doi: 10.1214/ss/1177013604.

[6] P. Bahrmann *et al.*, ‘Prognostic value of different biomarkers for cardiovascular death in unselected older patients in the emergency department’, *Eur. Heart J. Acute Cardiovasc. Care*, vol. 5, no. 8, pp. 568–578, Dec. 2016, doi: 10.1177/2048872615612455.

[7] A. Mayr and M. Schmid, ‘Boosting the Concordance Index for Survival Data – A Unified Framework To Derive and Evaluate Biomarker Combinations’, *PLOS ONE*, vol. 9, no. 1, p. e84483, Jan. 2014, doi: 10.1371/journal.pone.0084483.

[8] M. Schmid and T. Hothorn, ‘Boosting additive models using component-wise P-Splines’, *Comput. Stat. Data Anal.*, vol. 53, no. 2, pp. 298–311, Dec. 2008, doi: 10.1016/j.csda.2008.09.009.

[9] T. Kneib, T. Hothorn, and G. Tutz, ‘Variable Selection and Model Choice in Geoadditive Regression Models’, *Biometrics*, vol. 65, no. 2, pp. 626–634, 2009, doi: 10.1111/j.1541-0420.2008.01112.x.

[10] B. Hofner, A. Mayr, N. Robinzonov, and M. Schmid, ‘Model-based boosting in R: a hands-on tutorial using the R package mboost’, *Comput. Stat.*, vol. 29, no. 1–2, pp. 3–35, Feb. 2014, doi: 10.1007/s00180-012-0382-5.

[11] A. Mayr, B. Hofner, and M. Schmid, ‘The Importance of Knowing When to Stop: A Sequential Stopping Rule for Component-wise Gradient Boosting’, *Methods Inf. Med.*, vol. 51, no. 02, pp. 178–186, 2012, doi: 10.3414/ME11-02-0030.

[12] ‘Stability selection - Meinshausen - 2010 - Journal of the Royal Statistical Society: Series B (Statistical Methodology) - Wiley Online Library’. https://rss.onlinelibrary.wiley.com/doi/10.1111/j.1467-9868.2010.00740.x (accessed Oct. 22, 2021).

[13] R. D. Shah and R. J. Samworth, ‘Variable selection with error control: another look at stability selection: *Another Look at Stability Selection*’, *J. R. Stat. Soc. Ser. B Stat. Methodol.*, vol. 75, no. 1, pp. 55–80, Jan. 2013, doi: 10.1111/j.1467-9868.2011.01034.x.
