# Supplementary material for: Proteomics biomarker discovery for individualized prevention of familial pancreatic cancer using statistical learning
Source: PLoS One. 2023 Jan 26;18(1):e0280399. doi: 10.1371/journal.pone.0280399 (PMC9879447; doi:10.1371/journal.pone.0280399)
Supplement: S2 Appendix — (DOCX) [file pone.0280399.s011.docx]

S2 Appendix - Rmarkdown of data analysis in simulation study and FaPaCa study

## Simulation study

In this session, we are presenting the simulation study procedures in ***Proteomics biomarker discovery for individualized prevention of familial pancreatic cancer using statistical learning.***

For all simulations, we used the following data generating mechanism, mentioned in the above paper (DGM1):

$$\begin{matrix} f\sim N(0,1) \\ y\mid f\sim Bernoulli\left( \pi=invlogit\left( \tau+\sigma\cdot f \right) \right) \\ x_{j}\mid f\sim N\left( \sqrt{\rho_{j}}f, 0-\rho_{j} \right), for j=1,\ldots,p_{ref} \\ x_{j}\mid f\sim N\left( 0,1 \right), for j=p_{ref},\ldots,P \end{matrix}$$

## data simulator ######

binary_data_generator <- function(N, P, p_ref, tau, sigma, rho, min_n, link = "logit"){
 linkinv <- make.link(link)$linkinv

 f <- rnorm(N, 0, 1)
 y <- rbinom(N, 1, linkinv(tau + sigma*f))

 # regenerate if total number of minor group smaller than min_n
 while(sum(y) < min_n){
 y <- rbinom(N, 1, linkinv(tau + sigma*f))
 }

 x <- cbind(sqrt(1 - rho) * matrix(rnorm(N*p_ref, 0, 1), nrow = N) +
 sqrt(rho) * matrix(rep(f, p_ref), nrow = N),
 matrix(rnorm(N * (P - p_ref), 0, 1), nrow = N))

 return(list(y = y, x = x))
}

The following packages are used in this simulation:

## load required libraries ####
if(!require(data.table)) install.packages("data.table")
if(!require(purrr)) install.packages("purrr")
if(!require(magrittr)) install.packages("magrittr")
if(!require(caret)) install.packages("caret")
if(!require(glmnet)) install.packages("glmnet")
if(!require(doParallel)) install.packages("doParallel")
if(!require(foreach)) install.packages("foreach")
if(!require(mboost)) install.packages("mboost")

## Comparison of prediction performance

In the first simulation, we compare the prediction performance among ridge regression, adaptive lasso and statistical boosting (mboost).

### Simulating data

First, we use DGM1 to generate 100 training ($N=30$) and test ($N=20$) datasets respectively by setting the hyperparameter $P=500$, $p_{ref}=10$, $\tau=-8.5$, $\sigma=10$ and $\rho=\{0.05, 0.15,\ldots, 0.85, 0.95\}$

# for the purpose of reproducibility
set.seed(1118)

## generate binary data ####
simulated_train <- map(1:100, function(x)
 {
 simdat <- binary_data_generator(N = 30, P = 500, p_ref = 10,
 tau = -8.5, sigma = 10,
 rho = seq(0.05, 0.95, 0.1), min_n = 3)
 simdat <- data.table(as.factor(simdat$y), simdat$x)
 setnames(simdat, c("y", paste0("X", 1:500)))

 return(simdat)
 }
)

simulated_test <- map(1:100, function(x)
 {
 simdat <- binary_data_generator(N = 20, P = 500, p_ref = 10,
 tau = -8.5, sigma = 10,
 rho = seq(0.05, 0.95, 0.1), min_n = 3)
 simdat <- data.table(as.factor(simdat$y), simdat$x)
 setnames(simdat, c("y", paste0("X", 1:500)))

 return(simdat)
 }
)

### Rescaling datasets

Second, for each pair of training and test dataset, we standardize the training set and rescale the test set correspondingly.

vars <- paste0("X", 1:500)

## obtain means and standard deviation of each variable in each training set ####
train_mu_sd <- map(simulated_train, function(x)
 {
 return(list(mu = apply(x[, ..vars], 2, mean),
 std = apply(x[, ..vars], 2, sd)))
 }
)

## standardise the training datasets ####
simulated_train_rescaled <- map2(simulated_train, train_mu_sd,
 function(x, y)
 {
 return(cbind(x[, .(y)],
 scale(x[, ..vars],
 center = y$mu,
 scale = y$std)))
 })

## rescale the test datasets ####
simulated_test_rescaled <- map2(simulated_test, train_mu_sd,
 function(x, y)
 {
 return(cbind(x[, .(y)],
 scale(x[, ..vars],
 center = y$mu,
 scale = y$std)))
 })

### Model fitting

#### **Ridge regression**

- Ridge regression is fitted to each training dataset and the hyperparameter $\lambda$ is tuned via 5-fold cross validation.

## make cluster ####
cl <- makeCluster(detectCores())
registerDoParallel(cl)

## perform ridge regression ####
ridge_fit <- foreach(i = 1:100, .packages = "glmnet") %dopar% {

 # convert data to matrix
 xmat <- as.matrix(simulated_data_train[[i]][, -1])
 y <- simulated_data_train[[i]]$y

 # fit ridge regression
 glmnet::cv.glmnet(x = xmat, y = y,
 alpha = 0,
 nlambda = 200,
 family = "binomial",
 type.measure = "deviance",
 nfolds = 5)
}

## stop cluster ####
stopCluster(cl)

#### **Adaptive lasso**

- For each training data set, the adaptive penalty weights are first estimated using the variable coefficients of the optimal ridge model. The hyperparameter $\lambda$ is estimated via 5-fold cross validation

## make cluster ####
cl <- makeCluster(detectCores())
registerDoParallel(cl)

## perform adaptive lasso ####
adalasso_fit <- foreach(i = 1:100, .packages = c("glmnet", "caret")) %dopar% {

 # convert data to matrix
 xmat <- as.matrix(simulated_data_train[[i]][, -1])
 y <- simulated_data_train[[i]]$y

 # create the cross-validation
 foldid <- createFolds(as.factor(y), k = 5, list = FALSE)

 # estimate penalty factor (adpative weights) with ridge regression
 l2fit <- cv.glmnet(x = xmat, y = y,
 alpha = 0,
 nlambda = 200,
 family = "binomial",
 type.measure = "deviance",
 foldid = foldid)

 # obtain penalty factor
 weight <- as.vector(1/abs(coef(l2fit, s = "lambda.min")))[-1] # remove the intercept

 # fit adalasso
 cv.glmnet(x = xmat, y = y,
 alpha = 1,
 penalty.factor = weight,
 nlambda = 200,
 family = "binomial",
 foldid = foldid)

}

## stop cluster ####
stopCluster(cl)

#### **glmboost**

- The learning rate $\nu$ is set at $0.1$. The hyperparameter iteration $mstop$ is estimated according to the guideline of Mayr et al. 2012 that $mstop$ is first determined by AIC and then bootstrapping.

## make cluster ####
cl <- makeCluster(detectCores())
registerDoParallel(cl)

## fit glmboost ####
simdata_glmboost_list <- foreach(i = 1:100, .packages = "mboost") %dopar% {

 # setting initial number of iteration
 iter <- 50

 # fit the logistic boosting model
 fit <- glmboost(y ~ .,
 data = simulated_data_train[[i]],
 family = Binomial(link = "logit"),
 control = boost_control(mstop = iter, nu = 0.1))

 # increase the number of iteration until the optimal iteration according to AIC
 aic <- AIC(fit, method = "classical")

 # the maximum mstop is restricted at 1000
 while(iter <= mstop(aic) * 1.2 && iter < 1000){
 iter <- iter + 50
 mstop(fit) <- iter
 aic <- AIC(fit, method = "classical")
 }

 # set a resampling scheme.
 rsmp <- cv(model.weights(fit),
 type = "bootstrap",
 strata = fit$response)

 # use resampling to search for the optimal iteration
 mboost_cvrisk <- cvrisk(fit,
 folds = rsmp,
 mc.cores = 10)

 # obtain the optimal model
 mstop(fit) <- mstop(mboost_cvrisk)

 # return fitted model
 fit
}

## stop cluster ####
stopCluster(cl)

### Test prediction results

- At last, we obtain the prediction results of all test sets for each model.

## obtain test result ####
ridge_testpred <- rbindlist(
 map2(ridge_fit, simulated_data_test,
 function(x, y){
 data.table(y = as.factor(y$y),
 response = as.vector(predict(x,
 s = x$lambda.min,
 newx = as.matrix(y[, -1]),
 type = "response")),
 predict = factor(predict(x,
 s = x$lambda.min,
 newx = as.matrix(y[, -1]),
 type = "class"),
 levels = c(0, 1)))
 }))

adalasso_testpred <- rbindlist(
 map2(adalasso_fit, simulated_data_test,
 function(x, y){
 data.table(y = as.factor(y$y),
 response = as.vector(predict(x,
 s = x$lambda.min,
 newx = as.matrix(y[, -1]),
 type = "response")),
 predict = factor(predict(x,
 s = x$lambda.min,
 newx = as.matrix(y[, -1]),
 type = "class"),
 levels = c(0, 1)))
 }))

glmboost_testpred <- rbindlist(
 pmap(list(simdata_glmboost_list, simulated_data_test, 1:100),
 function(x,y,z) data.table(Simultaion = z,
 y = y$y,
 response = as.vector(predict(x,
 newdata = y,
 type = "response")))
 ))

## Identifying stable variables

- In this simulation study, we compare the ability of identifying relevant variables between stability selection using adaptive lasso and glmboost.

### Generating data

- We generate multiple datasets using the same simulator and the same configurations as in the first simulation study adjusting the parameters described as follows:

$$\begin{matrix} N=\{30, 50, 100\} \\ P=\{100, 500, 5000\} \\ p_{ref}=\{5, 10, 20\} \\ \tau=\{-4.5, -6.5, -8.5\} \\ \rho=\{low, mid, high\} \end{matrix}$$

- For each parameter adjustment, we only changed one parameter and kept the others fixed. For $\rho$, $low,mid,high$ represents the evenly distributed correlations within the intervals $[0.05, 0.35]$, $[0.35, 0.65]$ and $[0.65, 0.95]$, respectively. Thus, we generate 100 realizations for each scenario.

# generate data with various N
N <- rep(c(30, 50, 100), each = 100)

set.seed(39374)
simdata_N <- map(N, binary_data_generator,
 fn = 1, min_n = 3,
 P = 500, p_ref = 10, tau = -8.5,
 sigma = 10, rho = seq(0.05, 0.95, 0.1))

# generate data with various P
P <- rep(c(100, 500, 5000), each = 100)

set.seed(44234374)
simdata_P <- map(P, binary_data_generator,
 fn = 1, min_n = 3,
 N = 30, p_ref = 10, tau = -8.5,
 sigma = 10, rho = seq(0.05, 0.95, 0.1))

# generate data with various pref
pref <- rep(c(5, 10, 20), each = 100)

set.seed(123474)
simdata_pref <- map(pref,
 function(x){
 rho = seq(0.05, 0.95, length = x)
 binary_data_generator(N = 30, P = 500,
 p_ref = x, tau = -8.5,
 fn = 1, min_n = 3,
 sigma = 10, rho = rho)
 })

# generate data with various tau
tau <- rep(c(-4.5, -6.5, -8.5), each = 100)

set.seed(964896)
simdata_tau <- map(tau, binary_data_generator,
 N = 30, P = 500, p_ref = 10,
 fn = 1, min_n = 3,
 sigma = 10, rho = seq(0.05, 0.95, 0.1))

# generate data with various rho
rho <- rep(list(seq(0.05, 0.35, len = 10), # low
 seq(0.35, 0.65, len = 10), # mid
 seq(0.65, 0.95, len = 10)), # high
 each = 100)

set.seed(6418241)
simdata_rho <- map(rho,
 binary_data_generator,
 N = 30, P = 500, p_ref = 10,
 fn = 1, min_n = 3,
 sigma = 10, tau = -8.5)

### Complementary pairs stability selection

- Here, we create some helper functions to perform the complementary pairs stability selection.

## helper functions of adaptive lasso for stability selection####
glmnet.adalasso <- function(x, y, q, l2_lambda, type = c("conservative", "anticonservative"), family, gamma = 1, ...) {
 require(glmnet)

 # determine the penal factor
 ridgefit <- glmnet(x, y, alpha = 0, intercept = TRUE, family = family, ...)
 weight <- as.vector(1/abs(coef(ridgefit, s = l2_lambda))**gamma)[-1]
 pf <- ncol(x) * weight/sum(weight)

 ## fit model
 type <- match.arg(type)
 if (type == "conservative")
 fit <- suppressWarnings(glmnet(x, y, pmax = q, alpha = 1,
 family = family, penalty.factor = pf, ...))
 if (type == "anticonservative")
 fit <- glmnet(x, y, dfmax = q - 1, alpha = 1,
 family = family, penalty.factor = pf, ...)

 ## which coefficients are non-zero?
 selected <- predict(fit, type = "nonzero")
 selected <- selected[[length(selected)]]
 ret <- logical(ncol(x))
 ret[selected] <- TRUE
 names(ret) <- colnames(x)
 ## compute selection paths
 cf <- fit$beta
 sequence <- as.matrix(cf != 0)
 ## return both
 return(list(selected = ret, path = sequence))
}

## stability selection function with adaptive lasso ####
adalasso_cpss <- function(data, q = 20, PFER = 4){
 # stratified subsampling
 stabs_rsmp <- stabs::subsample(rep(1, nrow(data$x)), B = 50, strata = as.factor(data$y))

 simdata_stabsel <- stabs::stabsel(x = data$x, y = data$y,
 fitfun = glmnet.adalasso,
 args.fitfun = list(type = "conservative", family = "binomial", standardize = FALSE,
 l2_lambda = 10, weighted = FALSE, gamma = 1),
 sampling.type = "SS",
 assumption = "unimod",
 folds = stabs_rsmp,
 q = q, PFER = PFER)

 return(list(data = data, result = simdata_stabsel))
}

## stability selection function with glmboost ####
glmboost_cpss <- function(data, q = 20, PFER = 4){
 dimnames(data$x) <- list(NULL, paste0("X", 1:ncol(data$x)))

 # fit the logistic boosting model
 glmboost_fit <- glmboost(x = cbind(Intercept = 1, data$x),
 y = as.factor(data$y),
 family = Binomial(link = "logit"),
 control = boost_control(mstop = 500, nu = 0.1))

 # stratified subsampling
 stabs_rsmp <- subsample(model.weights(glmboost_fit), B = 50, strata = glmboost_fit$response)

 glmboost_stabsel <- stabsel(mboost_fit, q = q, PFER = PFER,
 sampling.type = "SS",
 assumption = "unimod",
 folds = stabs_rsmp,
 grid = 0:500)

 return(list(data = data, result = glmboost_stabsel))
}

#### CPSS with adaptive lasso

# perform stability selection with various N
adalasso_simulation_N <- foreach(i = 1:300) %do% {
 adalasso_cpss(simdata_N[[i]])
}

# perform stability selection with various P
adalasso_simulation_P <- foreach(i = 1:300) %do% {
 adalasso_cpss(simdata_P[[i]])
}

# perform stability selection with various pref
adalasso_simulation_pref <- foreach(i = 1:300) %do% {
 adalasso_cpss(simdata_pref[[i]])
}

# perform stability selection with various tau
adalasso_simulation_tau <- foreach(i = 1:300) %do% {
 adalasso_cpss(simdata_tau[[i]])
}

# perform stability selection with various rho
adalasso_simulation_rho <- foreach(i = 1:300) %do% {
 adalasso_cpss(simdata_rho[[i]])
}

#### CPSS with glmboost

# perform stability selection with glmboost
cl <- parallel::makeCluster(50)
doParallel::registerDoParallel(cl)

# perform stability selection with various N
glmboost_simulation_N <- foreach(i = 1:300, .packages = "mboost") %dopar% {
 glmboost_cpss(simdata_N[[i]])
}

# perform stability selection with various N
glmboost_simulation_P <- foreach(i = 1:300, .packages = "mboost") %dopar% {
 glmboost_cpss(simdata_P[[i]])
}

# perform stability selection with various N
glmboost_simulation_pref <- foreach(i = 1:300, .packages = "mboost") %dopar% {
 glmboost_cpss(simdata_pref[[i]])
}

# perform stability selection with various N
glmboost_simulation_tau <- foreach(i = 1:300, .packages = "mboost") %dopar% {
 glmboost_cpss(simdata_tau[[i]])
}

# perform stability selection with various N
glmboost_simulation_rho <- foreach(i = 1:300, .packages = "mboost") %dopar% {
 glmboost_cpss(simdata_rho[[i]])
}

parallel::stopCluster(cl)

## FaPaCa study

In this session, we are presenting the modelling procedures in the FaPaCa study in ***Proteomics biomarker discovery for individualized prevention of familial pancreatic cancer using statistical learning.***

The following packages are used in the analysis:

## load required libraries ####
if(!require(data.table)) install.packages("data.table")
if(!require(tidyverse)) install.packages("tidyverse")
if(!require(caret)) install.packages("caret")
if(!require(mboost)) install.packages("mboost")
if(!require(furrr)) install.packages("furrr")

### Model validation

To perform the model validation, we used the repeated four-fold cross validation to divide the data into multiple training and test datasets. The training datasets are standardized before fitting to a model and the corresponding test datasets are hence rescaled accordingly.

## create repated 4-fold CV ####
set.seed(91623978)
cv_ind <- createMultiFolds(dat$Status, 4, 10)

# rescale the data according to the train data
data_rescaled <- map(cv_ind,
 function(x){
 ind <- unique(x)
 data <- copy(olink_LH)[, c(olinkID) := lapply(.SD, function(y) (y - mean(y[ind]))),
 .SDcols = c(olinkID)]
 data[, Age := (Age - mean(Age[ind]))]
 return(list(train = data[ind], test = data[-ind]))
 })

data_train <- map(LH_cv_rescaled, function(x) return(x$train))
data_test <- map(LH_cv_rescaled, function(x) return(x$test))

First, we fit the glmboost model (with linear base-learners only) to each training data. We use the logistic model as the fundamental model and set the learning rate $\nu=0.1$. According to the recommendation in Mayr et al. 2012, we first estimated the optimal iteration $mstop$ using AIC. Then, we apply the bootstrapping for tuning the $mstop$. The optimal $mstop$ is estimated via the lowest averaged out-of-bag errors.

# schedule a parallisation with 2 cores
plan(multicore, workers=2)

glmboost_fit <- future_map(.x = data_train,
 .options = furrr_options(seed = 1006613948),
 .f = function(x){

 data = x[, c("Status", "Sex", "Age", ..olinkID)]

 # setting initial number of iteration
 iter <- 50

 # fit the logistic boosting model
 fit <- glmboost(Status ~ .,
 data = data,
 family = Binomial(link = "logit"),
 control = boost_control(mstop = iter, nu = 0.1))

 # adjust the number of iteration using AIC (logit link only)
 aic <- AIC(fit, method = "classical")

 while(iter <= mstop(aic) * 1.2 && iter < 1000){
 iter <- iter + 50
 mstop(fit) <- iter
 aic <- AIC(fit, method = "classical")
 }

 # set a resampling scheme.
 rsmp <- mboost::cv(model.weights(fit),
 type = "bootstrap",
 strata = fit$response)

 # using resampling to search for the optimal iteration (mstop)
 fit_cvrisk <- mboost::cvrisk(fit,
 folds = rsmp,
 mc.cores = 10)

 # set the mstop to optimal mstop
 mstop(fit) <- mstop(fit_cvrisk)

 return(fit)
 })

Second, we fit the gamboost model (with linear base-learners and smooth p-spline base-learners) to each training data. We define the linear and centered smooth p-spline base learners. An extra base-learner for the intercept is created.

# create base-learner
blrns <- c(
 paste0("bols(", c(olinkID, "Age", "Sex"),
 ", intercept = FALSE)"), # centred
 paste0("bbs(", c(olinkID, "Age"),
 ", knots = 8, degree = 4, df = 1, center = TRUE)"), # centred
 "bols(Intercept, intercept = FALSE)"
)

fmla <- as.formula(paste0("Status ~ ", paste(blrns, collapse = "+")))

We apply the same configuration and procedure for searching the optimal iteration like above.

# schedule a parallisation with 2 cores
plan(multicore, workers=2)

gamboost_fit <- future_map(.x = data_train,
 .options = furrr_options(seed = 1006613948),
 .f = function(x){

 # setting initial number of iteration
 iter <- 50

 # fit the logistic boosting model
 fit <- gamboost(formula = fmla,
 data = cbind(x, Intercept = 1),
 family = Binomial(link = "logit"),
 control = boost_control(mstop = iter, nu = 0.1))

 # adjust the number of iteration using AIC (logit link only)
 aic <- AIC(fit, method = "classical")

 while(iter <= mstop(aic) * 1.2 && iter < 1000){
 iter <- iter + 50
 mstop(fit) <- iter
 aic <- AIC(fit, method = "classical")
 }

 # set a resampling scheme.
 rsmp <- mboost::cv(model.weights(fit),
 type = "bootstrap",
 strata = fit$response)

 # using resampling to search for the optimal iteration.
 fit_cvrisk <- mboost::cvrisk(fit,
 folds = rsmp,
 mc.cores = 10)

 # set the mstop to optimal mstop
 mstop(fit) <- mstop(fit_cvrisk)

 return(fit)
 })

### Stability selection

We first pre-specify a large enough $\mathrm{mstop}$, such as 2000, and fit glmboost to the whole dataset. Hence, we subsample the dataset $B=50$ times. In stability selection, we set the number of base-learners included in each fitted model $q=10$ and the per-family error rate $PFER=2$.

## setting initial number of iteration ####
iter <- 2000

## fit the logistic boosting model ####
glmboost_fit <- glmboost(Status ~ .,
 data = data[, c("Status", "Sex", "Age", ..olinkID)],
 family = Binomial(link = "logit"),
 control = boost_control(mstop = iter, nu = 0.1))

set.seed(645332)
## startified subsampling ####
stabs_rsmp <- subsample(model.weights(glmboost_fit), B = 50, strata = glmboost_fit$response)

## stability selection ####
glmboost_stabsel <- stabsel(glmboost_fit, q = 10, PFER = 2,
 sampling.type = "SS",
 folds = stabs_rsmp,
 grid = 0:2000,
 assumption = "unimod",
 mc.cores = 10,
 eval = TRUE)

We repeat the same procedure using the similar configurations, except $q=20$ and $PFER=4$, with gamboost.

## setting initial number of iteration ####
iter <- 2000

## fit the logistic boosting model ####
gamboost_fit <- gamboost(formula = fmla,
 data = cbind(data, Intercept = 1),
 family = Binomial(link = "logit"),
 control = boost_control(mstop = iter, nu = 0.1))

set.seed(645332)
## startified subsampling ####
stabs_rsmp <- subsample(model.weights(gamboost_fit), B = 50, strata = gamboost_fit$response)

## stability selection ####
gamboost_stabsel <- stabsel(gamboost_fit, q = 20, PFER = 4,
 sampling.type = "SS",
 folds = stabs_rsmp,
 grid = 0:2000,
 assumption = "unimod",
 mc.cores = 10,
 eval = TRUE)
