# Supplementary material for: Proteomics biomarker discovery for individualized prevention of familial pancreatic cancer using statistical learning
Source: PLoS One. 2023 Jan 26;18(1):e0280399. doi: 10.1371/journal.pone.0280399 (PMC9879447; doi:10.1371/journal.pone.0280399)
Supplement: Attachment — Submitted filename: Response to reviewers.docx [file pone.0280399.s012.docx]

**Response to Reviewers**

**Journal Requirements:**

To Academic Editor: We would like to thank Prof. Batra for his effort in managing our submission and helpful comments. We have addressed the questions raised by Prof. Batra as follows:

1. Please ensure that your manuscript meets PLOS ONE's style requirements, including those for file naming. The PLOS ONE style templates can be found at

<https://journals.plos.org/plosone/s/file?id=wjVg/PLOSOne_formatting_sample_main_body.pdf>  and

<https://journals.plos.org/plosone/s/file?id=ba62/PLOSOne_formatting_sample_title_authors_affiliations.pdf>

Authors’ reply:

Thank you for the kind reminder. We have aligned our manuscript according to the PLOS ONE’s style requirements.

2. In your Data Availability statement, you have not specified where the minimal data set underlying the results described in your manuscript can be found. PLOS defines a study's minimal data set as the underlying data used to reach the conclusions drawn in the manuscript and any additional data required to replicate the reported study findings in their entirety. All PLOS journals require that the minimal data set be made fully available. For more information about our data policy, please see <http://journals.plos.org/plosone/s/data-availability>.

Upon re-submitting your revised manuscript, please upload your study’s minimal underlying data set as either Supporting Information files or to a stable, public repository and include the relevant URLs, DOIs, or accession numbers within your revised cover letter. For a list of acceptable repositories, please see <http://journals.plos.org/plosone/s/data-availability#loc-recommended-repositories>. Any potentially identifying patient information must be fully anonymized.

Important: If there are ethical or legal restrictions to sharing your data publicly, please explain these restrictions in detail. Please see our guidelines for more information on what we consider unacceptable restrictions to publicly sharing data: <http://journals.plos.org/plosone/s/data-availability#loc-unacceptable-data-access-restrictions>. Note that it is not acceptable for the authors to be the sole named individuals responsible for ensuring data access.

We will update your Data Availability statement to reflect the information you provide in your cover letter.

Authors’ reply:

We thank the academic editor for highlighting the insufficient information in the submitted Data Availability Statement. As mentioned in our cover letter, we would like to include the Data Availability Statement in our manuscript as follows:

***The individual-level data of the FaPaCa study are not publicly available, because the data contain sensitive patient data which underlie data protection rules. This is in accordance with the local ethic vote and the regulations of the FaPaCa registry. Patients’ characteristics are available upon request from the FaPaCa study registry (contact information: fapaca@med.uni-marburg.de). The aggregated results from the FaPaCa study and the full codes for the simulation are available in its Supporting Information files.***

3. Thank you for stating the following financial disclosure:

“This research was supported by a grant from the Wilhelm Sander-Stiftung to EPS, DKB, and KS (No. 2018.022.1) and a generous donation from the GAUFF-Foundation. Furthermore, this research was supported within the Munich Center of Health Sciences (MC-Health), Ludwig Maximilian University of Munich, as part of LMUinnovativ.”

Please state what role the funders took in the study.  If the funders had no role, please state: "The funders had no role in study design, data collection and analysis, decision to publish, or preparation of the manuscript."

If this statement is not correct you must amend it as needed.

Please include this amended Role of Funder statement in your cover letter; we will change the online submission form on your behalf.

Authors’ reply:

Thank you for reviewing the financial disclosure and requesting the role of the funders in the study. We have written the Role of Funder statement as follows:

***The funders had no role in study design, data collection and analysis, decision to publish, or preparation of the manuscript.***

This statement has been included in the cover letter.

4. Please include your full ethics statement in the ‘Methods’ section of your manuscript file. In your statement, please include the full name of the IRB or ethics committee who approved or waived your study, as well as whether or not you obtained informed written or verbal consent. If consent was waived for your study, please include this information in your statement as well.

Authors’ reply:

Thank you for the reminder of including our full ethics statement. We have included the full ethics statement in the “Materials and Methods” section (line 142– 144), written as follows:

***The FaPaCa registry, including the genetic analyses and the screening program, was approved by the Ethics Committee of the Philipps University of Marburg (No. 36/1997, last amendment 9/2010), and all participants provided written informed consent.***

5. Please include a copy of Table 1 which you refer to in your text on page 16.

Authors’ reply:

Thank you for the reminder of including a copy of Table 1. We have attached the Table 1 in the manuscript.

**Reviewers' comments:**

To Reviewer: We would like to thank the reviewer for the effort of reading and reviewing our manuscript. Also, we appreciate the reviewer’s comments and suggestions, providing an insightful perspective on the biological interpretation of our findings and helping us improve the quality of the manuscript. Therefore, we have responded to the reviewer’s comments and critique as follows:

1. Is the manuscript technically sound, and do the data support the conclusions?

The manuscript must describe a technically sound piece of scientific research with data that supports the conclusions. Experiments must have been conducted rigorously, with appropriate controls, replication, and sample sizes. The conclusions must be drawn appropriately based on the data presented.

Reviewer #1: Yes

2. Has the statistical analysis been performed appropriately and rigorously?

Reviewer #1: I Don't Know

Authors’ reply:

Thank you for sharing your opinions on our statistical analysis. The analysis approach we used in the manuscript is a statistical learning method - model-based boosting. It helped us to identify useful prognostic biomarkers in comprehensive protein profiles obtained by high-throughput proteomics. Additionally, we implemented another statistical method - stability selection - to identify a set of statistically stable biomarkers among the above-mentioned useful prognostic biomarkers. The set of statistically stable biomarkers refers to the set of biomarkers with a robust prognostic power. The technical details of the statistical modelling are demonstrated in the Rmarkdown file attached in the Supporting Information (S2 Appendix).

3. Have the authors made all data underlying the findings in their manuscript fully available?

The [PLOS Data policy](http://www.plosone.org/static/policies.action#sharing) requires authors to make all data underlying the findings described in their manuscript fully available without restriction, with rare exception (please refer to the Data Availability Statement in the manuscript PDF file). The data should be provided as part of the manuscript or its supporting information, or deposited to a public repository. For example, in addition to summary statistics, the data points behind means, medians and variance measures should be available. If there are restrictions on publicly sharing data—e.g. participant privacy or use of data from a third party—those must be specified.

Reviewer #1: No

Authors’ reply:

Thank you for pointing out that we did not provide the data underlying the finds in our manuscript. Owing to medical privacy and sensitive patient information, the data of the FaCaPa study are not publicly available, but patients’ characteristics are available upon request. Instead, the data in simulation study are available in the supporting information files. The data availability statement in the manuscript has been updated as follows:

***The individual-level data of the FaPaCa study are not publicly available, because the data contain sensitive patient data which underlie data protection rules. This is in accordance with the local ethic vote and the regulations of the FaPaCa registry. Patients’ characteristics are available upon request from the FaPaCa study registry (contact information: fapaca@med.uni-marburg.de). The aggregated results from the FaPaCa study and the full codes for the simulation are available in its Supporting Information files.***

4. Is the manuscript presented in an intelligible fashion and written in standard English?

PLOS ONE does not copyedit accepted manuscripts, so the language in submitted articles must be clear, correct, and unambiguous. Any typographical or grammatical errors should be corrected at revision, so please note any specific errors here.

Reviewer #1: Yes

5. Review Comments to the Author

Please use the space provided to explain your answers to the questions above. You may also include additional comments for the author, including concerns about dual publication, research ethics, or publication ethics. (Please upload your review as an attachment if it exceeds 20,000 characters)

Reviewer #1: This reviewer has expertise only in biological aspects of the manuscript. Biological part seems to be very confusing, couple biomarkers are mentioned, others are not. There is no table summarizing all stable biomarkers discovered by O-Link.

Authors’ reply:

Thank you for commenting on our manuscript in biological aspects and mentioning our unclear explanations in the biological part.

First, we would like to emphasize and clarify the concept of stable biomarkers. In our manuscript, stable biomarkers refer to the statistically stable biomarkers discovered by our statistical models, instead of biologically stable biomarkers.

We have created a summary table, including all biomarkers obtained via Olink and the corresponding resulting selection frequency in the stability selection analysis. All stable biomarkers have been asterisked in the table. The table has been attached in this document and in the Supporting Information, labelled as S1 Table.

20-biomarker panels are mentioned, but these biomarkers are not shown.

Authors’ reply:

The summary of selected biomarkers referring to Figure 6 is now also attached in this document and in the Supplementary Information, labelled as S2 Table.

In the introduction TIMP1 and LCN2 are mentioned. Were these biomarkers discovered by O-Link?

Authors’ reply:

As described in the corresponding publications Slater et al. 2013 and Bartsch et al. 2018, TIMP1 and LCN2 were discovered via the enzyme-linked immunosorbent assay (ELISA), instead of Olink.

What were other stable biomarkers discovered by O-Link, and what biological pathways do they represent? The biological part needs to be re-written for clarity and to better delineate biological significance of stable biomarkers.

Authors’ reply:

Thank you for mentioning the insufficient biological part about biological pathways and significance of discovered stable biomarkers. We have performed an overrepresentation analysis using the Reactome pathway database on the stable biomarkers (FGF-BP1, MSLN, PCSK9, PLA2G7 and LYPD3). The results are summarized in S3 Table in the manuscript, also attached in this document.  We have also rephrased the biological part (line 455 - 473 on p.17 & 18) in the manuscript, and addressed the stable biomarkers’ biological significance, shown as follows:

**Biological relevance of found stable biomarkers**

***Some previous studies showed that the discovered stable biomarkers in the FaPaCa study play a relevant role on various cancer developments. First, FGF-BP1 plays a crucial role on regulatory factors through binding directly to FGF-1 and FGF-2. Therefore, it boosts their biological activities, such as signaling, cell proliferation and angiogenesis [35], [36]. Furthermore, an overexpression of FGF-BP1 was found in pancreatic and colon cancer [37]. Second, MSLN was identified as a tumor-differentiation antigen with an overexpression discovered in multiple cancer types, such as epithelial mesothelioma, pancreatic cancer, and lung adenocarcinoma. It was shown to be a prognostic biomarker of early cancer-specific mortality of PDAC [38]. Third, Vainio et al. found that PLA2G7 promotes the cell migration of prostate cancer [39]. Fourth, some studies discussed the association of LYPD3 with progression of PDAC, melanoma and non-small cell lung cancer [40]******–[43]. Finally, PCSK9 may have an indirect influence on cancer growth via its cholesterol-regulating function, as the low-density-lipoprotein cholesterol supports tumor development [44], [45]. Li et al. also proposed that immune checkpoint therapy for cancer may benefit from inhibition of PCSK9 [46], [47]******.***

***In addition to the biological significance of individual biomarkers, we performed an over-representation analysis to explore the biological pathways of the discovered stable protein biomarkers. S3 Table shows 9 statistically significant biological pathways,*** ***i.e. adjusted p-value < 0.05, found in the Reactome pathway database [29]. Three of the pathways involve the stable biomarker pairs LYPD3-MSLN, providing convincing evidence on their close biological relationships and potential biological interaction.***

We also added the following paragraph to the Material and Methods (line 236 – 240 on p.9) section:

***To identify the biological pathway of the resulted subset of stable biomarkers, we performed an over-representation analysis using the Reactome pathway database [29]. In the analysis, all 330 eligible Oink biomarkers were used as background genes, but only 286 were recognized by Reactome. Fisher’s exact test was used to determine the statistical significance of the pathways and the Benjamini-Hochberg procedure was applied to control the false discovery rate. The significance level was set at 5%.***

We adjusted the section on Statistical software (line 244 – 247 on p.10) as follows:

***All statistical analyses were performed in the statistical programming language R (version 3.6.3) [30]. We performed ridge regression and adaptive lasso with package glmnet [31], mboost with package mboost [25], stability selection with package stabs [27], and over-representation analysis with package clusterProfiler and ReactomePA [32]–[34].***

6. PLOS authors have the option to publish the peer review history of their article ([what does this mean?](https://journals.plos.org/plosone/s/editorial-and-peer-review-process#loc-peer-review-history)). If published, this will include your full peer review and any attached files.

If you choose “no”, your identity will remain anonymous but your review may still be made public.

**Do you want your identity to be public for this peer review?** For information about this choice, including consent withdrawal, please see our [Privacy Policy](https://www.plos.org/privacy-policy).

Reviewer #1: No
