# Supplementary material for: Proteomics biomarker discovery for individualized prevention of familial pancreatic cancer using statistical learning
Source: PLoS One. 2023 Jan 26;18(1):e0280399. doi: 10.1371/journal.pone.0280399 (PMC9879447; doi:10.1371/journal.pone.0280399)
Supplement: Attachment — Submitted filename: 2nd_Revision_ResponseToReviwers.docx [file pone.0280399.s013.docx]

**Response to Reviewers**

**Reviewers' comments:**

To Reviewers:

We would like to thank the reviewers for the effort of reading and reviewing our manuscript. Also, we appreciate the reviewers’ comments and suggestions, providing an insightful perspective on cancer pathology and helping us improve the quality of the manuscript. Therefore, we have responded to the reviewer’s comments and critique as follows:

**Comments to the Author**

**1. If the authors have adequately addressed your comments raised in a previous round of review and you feel that this manuscript is now acceptable for publication, you may indicate that here to bypass the “Comments to the Author” section, enter your conflict of interest statement in the “Confidential to Editor” section, and submit your "Accept" recommendation.**

Reviewer #1: (No Response)

Reviewer #2: All comments have been addressed

2. Is the manuscript technically sound, and do the data support the conclusions?

The manuscript must describe a technically sound piece of scientific research with data that supports the conclusions. Experiments must have been conducted rigorously, with appropriate controls, replication, and sample sizes. The conclusions must be drawn appropriately based on the data presented.

Reviewer #1: No

Reviewer #2: Yes

3. Has the statistical analysis been performed appropriately and rigorously?

Reviewer #1: I Don't Know

Reviewer #2: Yes

4. Have the authors made all data underlying the findings in their manuscript fully available?

The PLOS Data policy requires authors to make all data underlying the findings described in their manuscript fully available without restriction, with rare exception (please refer to the Data Availability Statement in the manuscript PDF file). The data should be provided as part of the manuscript or its supporting information, or deposited to a public repository. For example, in addition to summary statistics, the data points behind means, medians and variance measures should be available. If there are restrictions on publicly sharing data—e.g. participant privacy or use of data from a third party—those must be specified.

Reviewer #1: Yes

Reviewer #2: No

5. Is the manuscript presented in an intelligible fashion and written in standard English?

PLOS ONE does not copyedit accepted manuscripts, so the language in submitted articles must be clear, correct, and unambiguous. Any typographical or grammatical errors should be corrected at revision, so please note any specific errors here.

Reviewer #1: Yes

Reviewer #2: Yes

6. Review Comments to the Author

Please use the space provided to explain your answers to the questions above. You may also include additional comments for the author, including concerns about dual publication, research ethics, or publication ethics. (Please upload your review as an attachment if it exceeds 20,000 characters)

Reviewer #1: This is a potentially significant study and access to unique FaPaCa samples is a great plus. However, the main premise of the study, i.e., 'Proteomics biomarker discovery for individualized prevention of familial pancreatic cancer' is not supported by the choice of samples. It looks like all samples for this study were coming from PDAC-free individuals. The presence of a pre-neoplastic lesion does not necessarily translate into cancer. The manuscript needs advice from a pancreatic oncologist.

Our reply:

Thank you very much for your review and advice. Our study aims to improve the detection of familial pancreatic cancer at its early, ideally pre-cancerous and still curable stage, with the help of protein biomarkers. Therefore, we focused on the individuals with precursor lesions, namely those with histologically significant lesions (HisSig), rather than individuals who already developed PDAC. High-grade pancreatic intraepithelial neoplasms (PanIN) and intraductal papillary-mucinous neoplasms (IPMN), which are well established obligate precancerous lesions (Ott et al. 2007, Permuth-Wey et al. 2009, Pittman et al. 2016, Makohon-Moore et al. 2018, Goggins et al. 2020), were found in the pancreas of the individuals in the HisSig group. Therefore, identifying and removing these lesions potentially can prevent the progression to PDAC. Since the visible pancreatic lesions on imaging cannot be reliably classified as benign or cancerous neither by endoscopic ultrasound (EUS) nor MRI, it is reasonable to assume that the IAR from FPC families and imagable pancreatic lesions (L group) are at risk for the development of PDAC in the future. We aim to provide an additional biomarker-based tool for regular screening of high-risk individuals to guide the decision for surgery with all the unfavourable consequences on life quality but with the major advantage of preventing the onset of PDAC. On the other hand, we agree that not all lesions progress to PDAC. Therefore, it is essential to identify novel biomarkers that can highlight persons with lesions on the path to progression to PDAC.

As a further aspect, we have opted to focus our analyses on individuals with precursor lesions rather than individuals with PDAC, since cancer development in the latter it is expected to have progressed to a more aggravating stage, at which already a larger portion of the proteomic and metabolic profile has been altered. Therefore, the results when contrasting patients with PDAC to unaffected individuals would most likely include biomarkers downstream of altered metabolic processes, e.g., caused by cachexia. (Dunne et al. 2022, Krapf et al. 2022, Shibata et al. 2022)

*References*

*Ott, C., Heinmöller, E., Gaumann, A. et al. Intraepitheliale Neoplasien (PanIN) und intraduktale papillär-muzinöse Neoplasien (IPMN) des Pankreas als Vorläufer des Pankreaskarzinoms. Med Klin 102, 127–135 (2007).* [*https://doi.org/10.1007/s00063-007-1013-8*](https://doi.org/10.1007/s00063-007-1013-8)

*Pittman ME, Rao R, Hruban RH. Classification, Morphology, Molecular Pathogenesis, and Outcome of Premalignant Lesions of the Pancreas. Arch Pathol Lab Med. 2017 Dec;141(12):1606-1614. doi: 10.5858/arpa.2016-0426-RA.*

*Makohon-Moore AP, Matsukuma K, Zhang M, Reiter JG, Gerold JM, Jiao Y, Sikkema L, Attiyeh MA, Yachida S, Sandone C, Hruban RH, Klimstra DS, Papadopoulos N, Nowak MA, Kinzler KW, Vogelstein B, Iacobuzio-Donahue CA. Precancerous neoplastic cells can move through the pancreatic ductal system. Nature. 2018 Sep;561(7722):201-205. doi: 10.1038/s41586-018-0481-8.*

*Goggins M, Overbeek KA, Brand R, Syngal S, Del Chiaro M, Bartsch DK, Bassi C, Carrato A, Farrell J, Fishman EK, Fockens P, Gress TM, van Hooft JE, Hruban RH, Kastrinos F, Klein A, Lennon AM, Lucas A, Park W, Rustgi A, Simeone D, Stoffel E, Vasen HFA, Cahen DL, Canto MI, Bruno M; International Cancer of the Pancreas Screening (CAPS) consortium. Management of patients with increased risk for familial pancreatic cancer: updated recommendations from the International Cancer of the Pancreas Screening (CAPS) Consortium. Gut. 2020 Jan;69(1):7-17. doi: 10.1136/gutjnl-2019-319352. Epub 2019 Oct 31. Erratum in: Gut. 2020 Jun;69(6):e3. PMID: 31672839; PMCID: PMC7295005.*

*Permuth-Wey, J., & Egan, K. M. (2009). Family history is a significant risk factor for pancreatic cancer: results from a systematic review and meta-analysis. Familial cancer, 8(2), 109–117. https://doi.org/10.1007/s10689-008-9214-8*

*Dunne RF, Roeland EJ. The Interplay Among Pancreatic Cancer, Cachexia, Body Composition, and Diabetes. Hematol Oncol Clin North Am. 2022 Oct;36(5):897-910. doi: 10.1016/j.hoc.2022.07.001.*

*Krapf SA, Lund J, Saqib AUR, Bakke HG, Rustan AC, Thoresen GH, Kase ET. Pancreatic Cancer Cell-Conditioned, Human-Derived Primary Myotubes Display Increased Leucine Turnover, Increased Lipid Accumulation, and Reduced Glucose Uptake. Metabolites. 2022 Nov 10;12(11):1095. doi: 10.3390/metabo12111095.*

*Shibata C, Otsuka M, Seimiya T, Kishikawa T, Ishigaki K, Fujishiro M. Lipolysis by pancreatic cancer-derived extracellular vesicles in cancer-associated cachexia via specific integrins. Clin Transl Med. 2022 Nov;12(11):e1089. doi: 10.1002/ctm2.1089.*

We thank the reviewer for the hint and have rephrased the 5th paragraph (line 84 - 94) in the section Background as follows and added the above-mentioned references:

*Since PDAC is often diagnosed at an advanced stage when patients show symptoms of major changes in metabolic processes, e.g. cachexia, proteomic and/or metabolic profiles of PDAC patients may already have been altered substantially [15]–[17]. Therefore, we aim to discover a robust subset of serum biomarkers for detection of significant lesions prior to or at an early, asymptomatic stage of cancer. To achieve this goal, we investigated individuals at risk (IARs) of FPC families with three different phenotypes: those without or with lesions detected on imaging by either magnetic resonance imaging (MRI) and/or endosonography, and those with histologically significant lesions. The latter include high-grade pancreatic intraepithelial neoplasms (PanIN) and intraductal papillary-mucinous neoplasms (IPMN) with dysplasia which are considered true precursor lesions of PDAC [12], [18]–[21]. Significant lesions, however, are rarely discovered in IARs, as they can only be confirmed histologically after the pancreatic surgery. This fact leads to small sample sizes and an unbalanced study design in the FaPaCa data.*

Reviewer #1 (continued): Identified biomarkers need to be validated in another set.

Our reply:

We agree that an independent set of samples is necessary to validate the discovered biomarkers further to generalize the findings presented in our work. However, given the currently available set of samples, such a validation would be beyond the scope of this work. Hence, to validate our findings to the best possible degree at least internally, we performed cross-validation within our samples to show the effectiveness of our statistical learning models and the discovered biomarkers in predicting the left-out individuals’ statuses. In this context, we also employed the statistical method termed stability selection. Since we could detect a stable set of biomarkers with high potential to improve diagnostics, we consider our findings and the statistical analysis guideline for handling the small unbalanced sample sets important for the community.

However, we see the point to address the issue of clinical validation and stability of the selected biomarker sets and rephrased the second part of the paragraph Strengths and Limitations (line 456 - 461) as follows:

*Hence, we reduced the flexibility of statistical learning methods to maintain the interpretability of the results and introduced an additional layer of stability selection to identify plausible and stable subsets of biomarkers. Furthermore, we conducted simulation studies for a thorough investigation of the validity and robustness of our findings. We consider our analyses to be an important hypothesis-generating step, to be succeeded by several subsequent clinical evaluation and validation procedures. For future research, it would be of interest to define a semi-supervised screening procedure that integrates prior clinical knowledge about protein networks, allows prioritizing variables within known clusters, and defines a subset of biomarkers that is hard-wired to be included in the model.*

Reviewer #2: The submitted manuscript by Ha et al applied advance simulation methods, like adaptive lasso to analyze the proteomic data to identify the promising biomarkers for the pancreatic cancer. The authors concluded that application of these advance learning methods improve the predictive accuracy of the proteomic data and provided the examples of FGF-BP1, PCSK9, and MSLN in pancreatic cancer inception and progression. Previous studies have also implicated FGF-BP1, and MSLN in pancreatic cancer pathology. Overall, the manuscript is well written and presented to understand the application of advance statistical methods. Moreover, authors addressed all the previous concerns.

Our reply:

Thank you very much for reviewing our paper and appreciating our results and statistical learning methods applied in our work.

7. PLOS authors have the option to publish the peer review history of their article (what does this mean?). If published, this will include your full peer review and any attached files.


If you choose “no”, your identity will remain anonymous but your review may still be made public.


Do you want your identity to be public for this peer review? For information about this choice, including consent withdrawal, please see our Privacy Policy.

Reviewer #1: No

Reviewer #2: No
